# DVM-SLC: A Dual-View Meta-Aware Model for Reliable Multi-Class Skin Lesion Classification from Clinical and Dermoscopic Images

**DOI:** 10.3390/diagnostics16162543

**Published:** 2026-08-12

**Authors:** Gökçen Çetinel, Sevda Gül, Rabia Öztaş Kara

**Affiliations:** 1 Electrical and Electronics Engineering Department, Engineering Faculty, Sakarya University, 54050 Serdivan, Türkiye; 2 Sakarya University Technology Development Zones Management Inc. (Sakarya Teknokent), Sakarya University Esentepe Campus, 54050 Serdivan, Türkiye; 3 Department of Electronics and Automation, Adapazarı Vocational School, Sakarya University, 54050 Serdivan, Türkiye; gulsevda@sakarya.edu.tr; 4 Department of Dermatology, Sakarya University Training and Research Hospital, 54050 Serdivan, Türkiye; rabiaoztaskara@sakarya.edu.tr

**Keywords:** skin lesion classification, dermoscopy, clinical imaging, dual-view learning, gated fusion, calibration, robustness, explainability

## Abstract

**Background/Objectives:** Skin lesion classification remains challenging in real clinical settings because diagnostically relevant information is distributed across different imaging modalities, class imbalance is often severe, and overall performance alone may conceal important weaknesses in model behavior. In this study, a dual-view meta-aware model, termed DVM-SLC was developed for multi-class skin lesion classification on the MILK10k dataset. **Methods:** The model jointly processes paired clinical and dermoscopic images and incorporates patient-level contextual information through a dedicated metadata branch. Cross-view information is integrated by a gated fusion mechanism to preserve modality-specific representations while allowing adaptive interaction between the two views. The model was evaluated not only in terms of comparative classification performance, but also with respect to calibration, robustness under common image corruptions, and expert-supported explainability. **Results:** Among the configurations evaluated using pooled out-of-fold predictions, the gated DVM-SLC achieved the highest Accuracy (0.7002) and the highest observed Macro-AUC (0.8774), whereas the gate-free dual-view model with metadata achieved the highest Macro F1-score (0.4174). The accuracy difference between the gated model and the dermoscopic-only baseline, which provided the highest non-proposed Accuracy, was statistically significant, whereas the observed Macro-AUC difference was not statistically significant. These findings indicated a metric-dependent trade-off rather than uniform superiority of a single fusion strategy. The raw confidence estimates were already reasonably aligned with empirical correctness, and temperature scaling provided only a modest, fold-dependent improvement in aggregate calibration. In robustness analysis, the strongest degradation was observed under Gaussian noise, while the model remained comparatively stable under blur, JPEG compression, brightness variation, contrast variation, and color shift. The explainability analysis was complemented by a structured dermatologist review. Gradient-weighted Class Activation Mapping (Grad-CAM) outputs from the clinical and dermoscopic branches were examined to determine whether the highlighted regions localized the lesion and corresponded to clinically meaningful morphological or dermoscopic features. This assessment was qualitative and was not intended as quantitative localization validation. **Conclusions:** Overall, the findings present DVM-SLC as a broadly evaluated approach with metric-dependent strengths and clear limitations, particularly in minority-class recognition. Its contribution lies in combining comparative performance analysis with calibration, robustness, and structured qualitative explainability assessment.

## 1. Introduction

Skin cancers are among the most frequently diagnosed malignancies worldwide and continue to pose a significant public health burden. According to global estimates from 2022, more than 1.5 million new skin cancer cases were reported within a single year. Among these, malignant melanoma, the most aggressive subtype, accounted for approximately 330,000 new cases and nearly 60,000 deaths worldwide. The incidence of melanoma exhibits substantial geographic variability and is often reported at higher rates in men than in women across many regions. These trends highlight the critical importance of early and accurate detection, as well as the growing need for imaging-based artificial intelligence (AI) systems that can operate reliably within clinical decision-support settings [1].

In routine dermatological practice, lesion assessment commonly relies on two complementary imaging modalities: clinical macroscopic photographs and dermoscopic images. Clinical images provide contextual information regarding lesion location, global morphology, and surrounding skin tissue, whereas dermoscopic images reveal diagnostically relevant microstructures such as pigment networks, vascular patterns, and fine textural details. Despite this complementary nature, a large proportion of existing deep learning–based approaches continue to operate on a single imaging modality, limiting their ability to integrate clinical and dermoscopic information in a balanced, sample-specific manner. Furthermore, real-world factors such as variations in illumination, camera devices, skin tone distribution, anatomical site diversity, and severe class imbalance introduce additional challenges that can adversely affect both predictive accuracy and generalizability.

These limitations become particularly evident in complex datasets such as MILK10k [2], which comprises 11 diagnostic classes and reflects real-world clinical conditions, including pronounced class imbalance and partial annotation uncertainty. While several dual-view and multi-modal learning strategies have been explored in the literature, frameworks that jointly analyze paired clinical and dermoscopic images together with patient metadata, while also examining calibration, robustness, and explainability, remain relatively scarce.

To address these gaps, this study develops a dual-view meta-aware framework, termed the Dual-View Meta-Aware Skin Lesion Classifier (DVM-SLC), for multi-class skin lesion classification. The contribution does not arise from any individual component in isolation, but from the controlled integration of paired clinical and dermoscopic representations, structured patient metadata, and a multi-stage evaluation protocol within the imbalanced 11-class MILK10k setting. The proposed model employs two separate EfficientNet-B3 backbones to extract modality-specific representations from clinical and dermoscopic images. Rather than relying on a fixed fusion strategy, these representations are integrated through a sample-adaptive gated fusion mechanism, allowing the model to dynamically emphasize the most informative modality for each individual case. In addition, patient metadata—including age, sex, anatomical region, and skin tone—are processed through a dedicated embedding subnetwork, enriching image-based representations with complementary clinical context.

The evaluation was designed to examine model behavior beyond classification performance alone. Model robustness is systematically assessed under a range of realistic image corruptions, while calibration analyses are conducted to quantify the reliability of predictive confidence. Moreover, explainable artificial intelligence (XAI) techniques are employed to enhance transparency: Gradient-weighted Class Activation Mapping (Grad-CAM) is used to visualize salient image regions influencing predictions, and SHapley Additive exPlanations (SHAP) analysis is applied to interpret the contribution of metadata features [3,4].

Accordingly, the system-level contribution of this study can be described through four connected components. First, it introduces a dual-view classification model that processes paired clinical and dermoscopic images through separate EfficientNet-B3 backbones and integrates the resulting representations by gated fusion. Second, it incorporates patient-level contextual information through a dedicated metadata branch, allowing lesion appearance to be interpreted together with clinically relevant auxiliary variables. Third, it evaluates the proposed model in terms of classification performance, calibration, and robustness under common image perturbations. Finally, it supports the explainability analysis with dermatologist-guided interpretation, making it possible to assess whether model attention remains clinically meaningful at the case level.

The study therefore examines how paired-view fusion, structured metadata, and reliability-focused evaluation operate together within an imbalanced 11-class setting. Its contribution is framed as a system-level integration and analysis rather than as the introduction of a new backbone, fusion primitive, or explainability method.

## 2. Related Work

Multi-class skin lesion classification has been studied extensively in recent years, largely because of its direct relevance to early diagnosis and clinical decision support [5,6,7,8,9,10,11,12]. Most existing studies have been developed on dermoscopic benchmark datasets such as HAM10000 and the International Skin Imaging Collaboration (ISIC) 2019 dataset, even though these datasets exhibit pronounced class imbalance and substantial overlap between visually similar categories [13,14,15,16,17]. Larger or aggregated datasets have also been used to increase diagnostic coverage [18,19,20,21,22,23]. However, across these settings, class imbalance remains a central problem, and minority categories continue to represent a persistent source of performance degradation.

To address these challenges, previous studies have investigated stronger convolutional architectures, ensemble learning, hybrid feature extraction, advanced augmentation, and imbalance-aware optimization. Ensemble-based models combining multiple convolutional backbones have frequently reported strong aggregate classification results [18,24,25]. Classical and hybrid feature-based approaches have also been used together with feature selection and machine learning classifiers [26,27]. More recently, balancing strategies, generative adversarial network-based augmentation, and transformer architectures have been proposed to improve recognition of underrepresented categories [28,29,30,31]. Multimodal fusion has additionally been explored to integrate clinical images with complementary information sources, including high-frequency ultrasound [32]. Collectively, these studies indicate that heterogeneous information can improve discrimination, although the magnitude and consistency of the benefit remain dependent on the dataset, fusion strategy, diagnostic formulation, and class distribution.

Recent multimodal skin lesion studies have examined more directly how clinical photographs, dermoscopic images, and patient information should be combined. FusionM4Net introduced a two-stage multimodal framework that integrates clinical and dermoscopic representations at the feature level and subsequently combines modality-specific outputs at the decision level [33]. Transformer-based single-stage fusion was later proposed to enable attention-mediated interaction between image and patient information while also providing modality-specific interpretability [34]. An alternative multi-task formulation combined metadata and handcrafted information through label-level fusion rather than conventional feature concatenation [35]. These approaches demonstrate that multimodal benefit depends not only on the inclusion of auxiliary information, but also on the stage at which different information sources interact and the representation used for each modality.

More recent work has moved toward deeper cross-modal interaction, adaptive fusion, and label-efficient multimodal learning. SkinM2Former uses tri-modal cross-attention to integrate clinical images, dermoscopic images, and structured metadata at multiple transformer feature levels while also modelling dependencies among diagnostic labels [36]. Self-supervised multimodal learning has been applied to paired clinical and dermoscopic images to exploit correspondence between the two views and reduce dependence on extensively annotated training data [37]. Cross-attention has also been used to fuse dermoscopic image features with patient metadata in combination with collaborative device–edge inference [38]. In another adaptive formulation, clinical images, dermoscopic images, and metadata were integrated through intermediate image fusion and uncertainty-guided late fusion [39]. Although these methods provide richer cross-modal interaction than fixed concatenation, most were evaluated under multi-label formulations, seven-point checklist tasks, or other experimental settings that differ from the imbalanced 11-class MILK10k problem considered here.

Medical vision–language models represent a related but methodologically distinct direction. MedSigLIP is a medically tuned SigLIP-based image–text encoder designed to align medical images with natural-language representations and to support applications such as classification and retrieval [40]. This form of large-scale image–text pretraining can provide transferable visual representations and semantic search capabilities. However, the input structure considered in the present study was different. No clinical reports, captions, or free-text descriptions were available, and the auxiliary information consisted of four structured variables. Although MedSigLIP could be adapted as a pretrained image encoder, converting the structured variables into textual prompts would introduce an additional representation and prompt-design stage. DVM-SLC instead preserves the native numerical and categorical forms of the metadata and integrates them directly with paired clinical and dermoscopic representations. The two approaches should therefore be regarded as complementary formulations rather than directly interchangeable metadata-processing strategies.

Beyond architectural design, the scope of model evaluation remains an additional concern in skin lesion analysis. A strong global score does not necessarily indicate reliable or clinically meaningful behavior. Several studies report high Accuracy or area under the receiver operating characteristic curve values [16,19,28], yet degradation in visually similar or underrepresented categories often becomes apparent when macro-averaged and class-wise metrics are examined. Calibration, corruption robustness, and expert-supported explainability also remain less consistently reported than aggregate classification performance. As a result, model strengths and weaknesses may not be fully visible when evaluation is limited to aggregate discrimination measures.

Against this background, DVM-SLC was designed to address several related gaps within a single experimental setting. The framework jointly processes paired clinical and dermoscopic images and incorporates age, sex, anatomical site, and skin tone through a dedicated structured metadata pathway. Its evaluation extends beyond aggregate discrimination to include class-wise behavior, statistical comparison, confidence calibration, corruption robustness, metadata ablation, computational characteristics, and dermatologist-supported explainability. The contribution of the present work is therefore positioned in the controlled integration and systematic evaluation of these components within an imbalanced 11-class MILK10k setting, rather than in a claim that gated fusion is universally superior to transformer-based, cross-attention, self-supervised, or vision–language alternatives.

## 3. Materials and Methods

**Figure 1:**
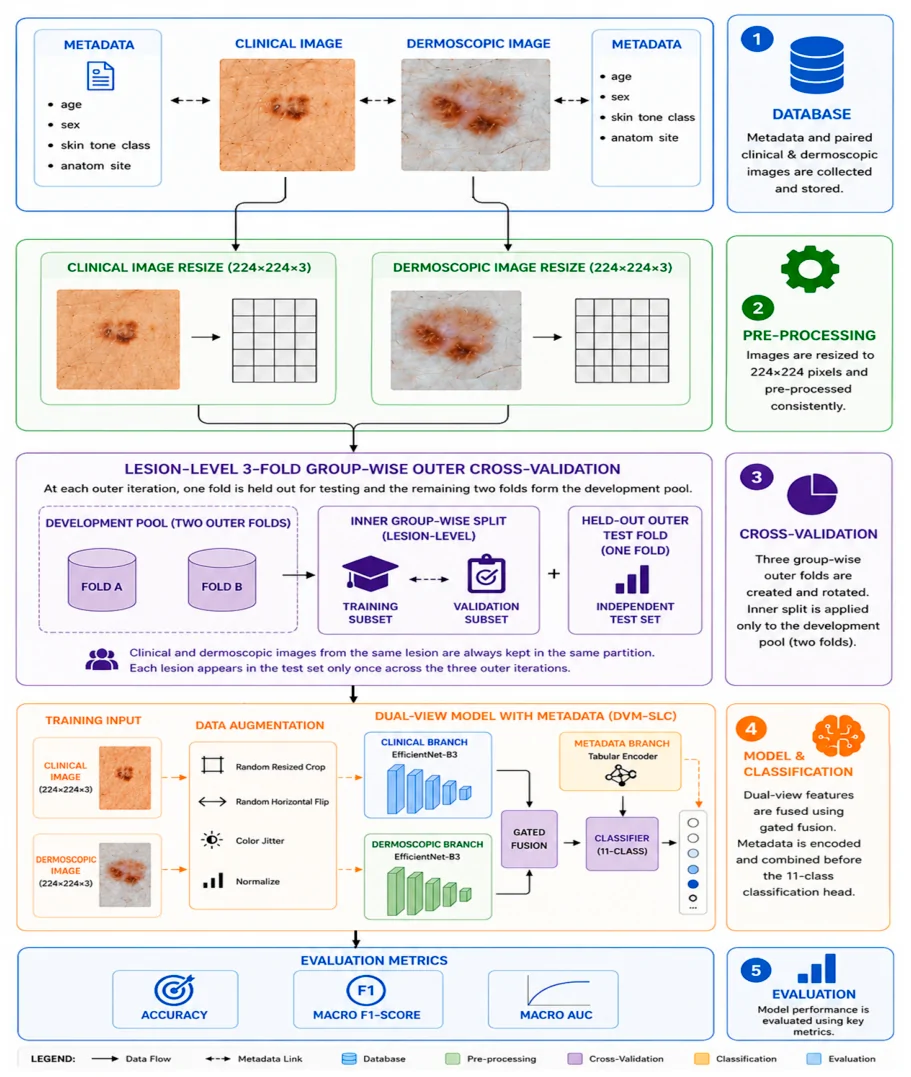
Overall workflow of the proposed study, showing paired clinical and dermoscopic inputs, preprocessing, lesion-level 3-fold group-wise cross-validation, dual-view classification with gated fusion, and performance evaluation.

This section describes the methodological framework developed for expert-validated dual-view skin lesion classification on the MILK10k dataset. The study was designed around three tightly connected components: the construction of a clinically realistic experimental setting based on paired clinical and dermoscopic images, the development of a dual-view and meta-aware classification framework, and the evaluation of this framework across complementary performance and reliability criteria. Accordingly, the following subsections present the dataset and study design, the proposed model architecture, and the experimental strategy used to assess classification performance, calibration behavior, robustness under common image corruptions, and explainability through both model-driven and expert-supported analyses. The overall workflow of the proposed study is summarized in Figure 1.

### 3.1. Dataset and Study Design

All experiments were conducted on the MILK10k dataset [2], a multi-center and multi-modal skin lesion imaging resource composed of paired clinical and dermoscopic images acquired from the same lesion. This paired structure was considered particularly valuable for the present study because clinical macroscopic photographs and dermoscopic images provide complementary diagnostic information in routine dermatological assessment. While clinical images preserve the global appearance of the lesion together with surrounding skin context, dermoscopic images reveal fine-scale structures that are often critical for differential diagnosis. By jointly analyzing both views, a more clinically realistic learning setting was established than would be possible with a single-modality formulation alone.

The dataset comprises 10,480 images corresponding to 5240 unique lesions. Among these lesions, 95.7% of the diagnostic labels were histopathologically confirmed, whereas the remaining cases were annotated based on expert dermatologist consensus. All labels were standardized according to the hierarchical diagnostic taxonomy used in the ISIC archive to ensure consistency across categories [2]. Rather than restricting the problem to binary benign-malignant discrimination, the present work addressed an 11-class classification setting. The 11 diagnostic categories are: actinic keratosis/intraepithelial carcinoma (AKIEC), basal cell carcinoma (BCC), squamous cell carcinoma/keratoacanthoma (SCCKA), melanoma (MEL), other malignant lesions (MAL_OTH), benign keratosis-like lesions (BKL), melanocytic nevus (NV), other benign lesions (BEN_OTH), vascular lesions (VASC), inflammatory lesions (INF), and dermatofibroma (DF). These abbreviations are used consistently throughout the study. This choice was made intentionally, since it better reflects real-world dermatological decision-making, where diagnostically relevant categories extend beyond a simple malignant-versus-benign separation and include lesions with substantial visual overlap and marked class imbalance.

To prevent data leakage, all data partitions were constructed at the lesion level, such that images originating from the same lesion were never allowed to appear in different subsets. Model development and evaluation were performed using a true 3-fold group-wise cross-validation protocol. In each run, one fold was used for testing, one for validation, and the remaining fold for training. Across the full protocol, each lesion appeared exactly once in the test set and once in the validation set, thereby enabling robust performance estimation while making efficient use of the available data. Fold construction was additionally constrained in a stratified group-wise manner so that all 11 diagnostic classes were represented in the test subset of each fold. Importantly, the native class imbalance of MILK10k was preserved throughout training and evaluation to better reflect real clinical prevalence rather than an artificially balanced research setting.

In addition to the paired images, age, sex, anatomical site, and skin tone were available as auxiliary inputs. Metadata preprocessing was performed independently within each cross-validation fold using parameters estimated exclusively from the corresponding training subset. Age and the ordinal skin-tone score were treated as numerical variables, whereas sex and anatomical site were treated as categorical variables. The processed metadata were incorporated after dual-view visual fusion through the feature-level pathway described in Section 3.2.

### 3.2. Proposed Framework

**Figure 2:**
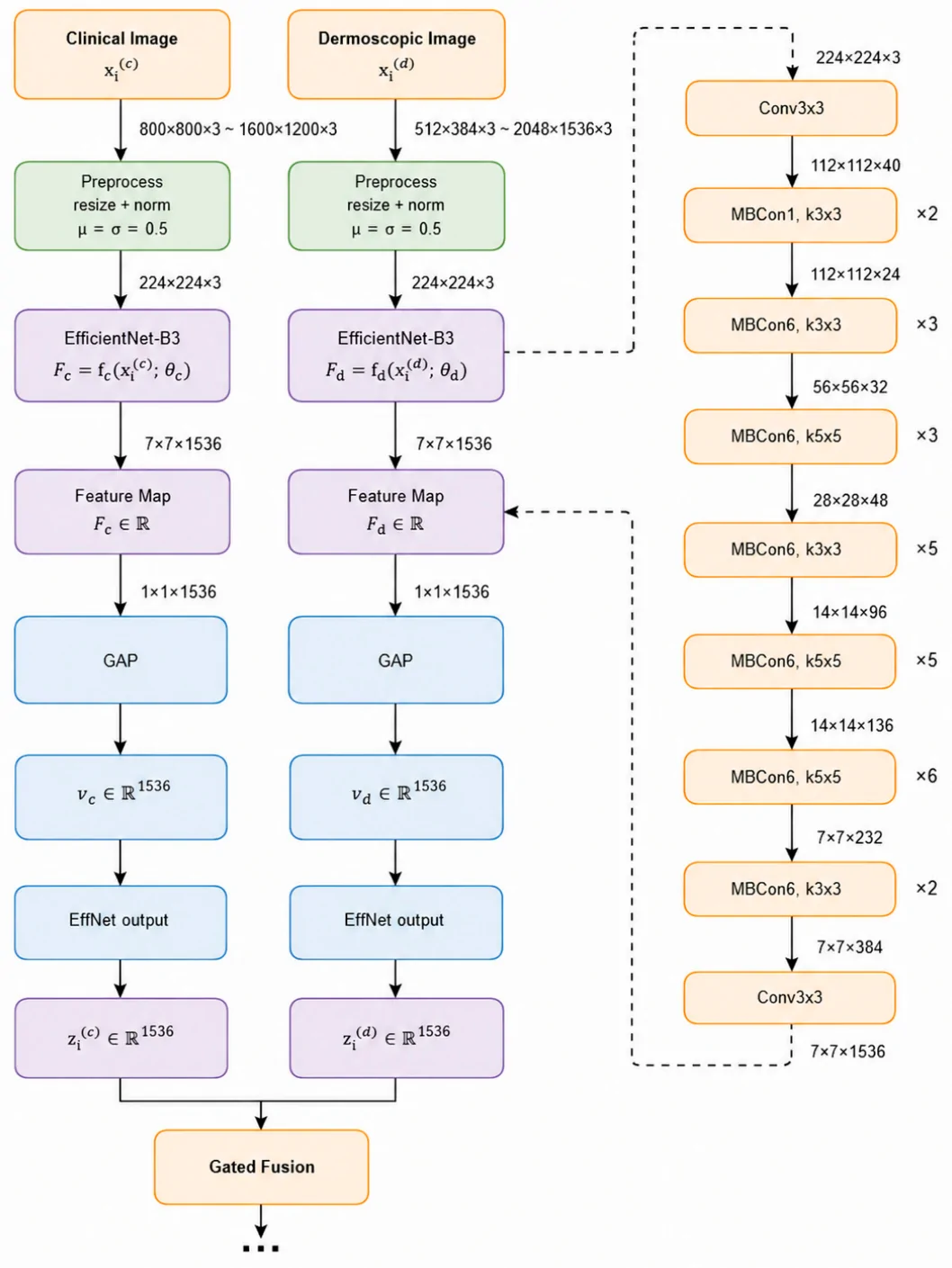
Visual architecture of the proposed dual-view framework, illustrating modality-specific preprocessing, EfficientNet-B3-based feature extraction, global average pooling, and gated fusion of clinical and dermoscopic representations. Metadata integration and the classification head are omitted from the figure for clarity and are formalized in Equations (8)–(11).

The proposed framework was developed to jointly analyze paired clinical and dermoscopic images while optionally incorporating patient-level metadata within a unified multi-class classification model. The visual backbone of the proposed dual-view framework is illustrated in Figure 2. In this design, modality-specific visual features are first extracted independently from clinical and dermoscopic inputs, then fused through a sample-adaptive gating mechanism, and finally combined with metadata-derived contextual information before classification. Formally, the proposed framework maps paired clinical and dermoscopic images, together with optional patient metadata, to an 11-class posterior probability distribution through a sequence of modality-specific feature extraction, adaptive fusion, contextual enrichment, and final classification.

For the i-th lesion sample, the input is defined as a paired multimodal tuple

(1) $$x_{i}=\left(x_{i}^{\left(c\right)}x_{i}^{\left(d\right)}m_{i}\right),$$

where $x_{i}^{\left(c\right)}\in R^{H\times W\times 3}$ denotes the clinical image, $x_{i}^{\left(d\right)}\in R^{H\times W\times 3}$ denotes the dermoscopic image, and $m_{i}\in R^{M}$ represents the optional metadata vector containing age, sex, anatomical localization, and skin tone. All images were resized to a fixed spatial resolution of H=W=224. During training, random resized cropping and random horizontal flipping were applied to both modalities to improve generalization. After augmentation, each image was normalized channel-wise using μ=(0.5,0.5,0.5) and σ=(0.5,0.5,0.5), resulting in inputs scaled to the range $\left[-1,1\right]$. No stochastic augmentation was applied during validation or testing.

To extract modality-specific representations, two independent EfficientNet-B3 encoders were employed [41], one for the clinical branch and one for the dermoscopic branch. EfficientNet-B3 was selected as a moderate-capacity convolutional backbone to keep the two-branch architecture computationally manageable while preserving separate feature extraction for the clinical and dermoscopic views. Using the same established backbone in both branches also allowed the study to focus on the effects of paired-view fusion and metadata integration without introducing additional variation from dissimilar visual encoders. This choice was not intended to imply superiority over more recent CNN or transformer-based backbones. A matched backbone comparison was not performed and should be addressed in future work. The modality-specific feature maps were defined as:

(2) $$F_{i}^{\left(c\right)}=f_{c}\left(x_{i}^{\left(c\right)}\right),F_{i}^{\left(d\right)}=f_{d}\left(x_{i}^{\left(d\right)}\right),$$

where $f_{c}(\cdot )$ and $f_{d}(\cdot )$ denote the feature extraction functions of the clinical and dermoscopic branches, respectively. The resulting feature maps were then transformed into compact global embeddings by global average pooling:

(3) $$z_{i}^{\left(c\right)}=GAP\left(F_{i}^{\left(c\right)}\right),z_{i}^{\left(d\right)}=GAP\left(F_{i}^{\left(d\right)}\right),$$

with $z_{i}^{\left(c\right)},z_{i}^{\left(d\right)}\in R^{D}.$ This two-branch structure was adopted to avoid premature parameter sharing between modalities that carry inherently different diagnostic information at macroscopic and dermoscopic scales.

The two modality-specific embeddings were integrated through a gated dual-view fusion strategy. First, the embeddings were concatenated as

(4) $$z_{i}^{\left(cat\right)}=\left[z_{i}^{\left(c\right)}z_{i}^{\left(d\right)}\right]\in R^{2D}.$$

A scalar gating coefficient was then computed by a lightweight two-layer perceptron followed by sigmoid activation:

(5) $$h_{i}=\phi \left(W_{1}z_{i}^{\left(cat\right)}b_{1}\right),g_{i}=\sigma \left(W_{2}h_{i}b_{2}\right),$$

where $\phi \left(\cdot \right)$ denotes the rectified linear unit (ReLU) activation and $\sigma \left(\cdot \right)$ is the sigmoid function. Here, W_1_ ∈ ℝ *^h^* ^× 2^**^D^** and W_2_ ∈ ℝ ^1 × h^ with hidden dimension h set to 1536, resulting in a compact gating network. The gate was used to reweight the two modality embeddings as

(6) $${\tilde{z}}_{i}^{\left(c\right)}=g_{i}z_{i}^{\left(c\right)},{\tilde{z}}_{i}^{\left(d\right)}=(1-g_{i})z_{i}^{\left(d\right)},$$

and the fused visual representation was obtained by concatenation:

(7) $$z_{i}^{\left(f\right)}=\left[{\tilde{z}}_{i}^{\left(c\right)}{\tilde{z}}_{i}^{\left(d\right)}\right]\in R^{2D}.$$

This mechanism allowed the network to adaptively emphasize the modality providing more reliable evidence for a given lesion instead of relying on a fixed fusion rule across all samples.

Patient-level metadata were incorporated through a dedicated feature-level pathway after the dual-view visual representation had been obtained according to Equation (7). The metadata associated with lesion i consisted of two numerical variables, age and the ordinal skin-tone score, and two categorical variables, sex and anatomical site. Within each cross-validation fold, age and skin tone were z-standardized using the mean and standard deviation estimated exclusively from the corresponding training subset. Sex and anatomical site were encoded using categorical mappings derived from the same training subset and were subsequently represented by learnable embedding vectors. All preprocessing operations fitted on the training subset were applied unchanged to the corresponding validation and test subsets.

(8) $$z_{i}^{\left(m\right)}=f_{m}\left(\left[{e_{i}^{\left(sex\right)};e_{i}^{\left(site\right)};\tilde{a}}_{i};{\tilde{t}}_{i}\right]\right)\in R^{64},$$

where $f_{m}\left(\cdot \right)$ denotes the metadata projection network, $e_{i}^{\left(sex\right)}$ and $e_{i}^{\left(site\right)}$ denote the learnable embeddings of sex and anatomical site for lesion i, respectively, and ${\tilde{a}}_{i}$ and ${\tilde{t}}_{i}$ denote the standardized age and skin-tone values. Equation (8) transforms the heterogeneous metadata components into a compact 64-dimensional representation. The metadata were therefore not projected into the pixel domain and were not required to have the same dimensionality as the visual representation.

(9) $$z_{i}^{\left(final\right)}=\left\{\begin{array}{c} \left[z_{i}^{\left(f\right)};z_{i}^{\left(m\right)}\right],\;when\;metadata\;are\;included.\\ z_{i}^{\left(f\right)},\;when\;metadata\;are\;excluded. \end{array}\right.$$

In Equation (9), $z_{i}^{\left(f\right)}$ is the dual-view visual representation obtained using the fusion mechanism defined in Equation (7), and $z_{i}^{\left(m\right)}$ is the metadata representation defined in Equation (8). When metadata were included, the two vectors were concatenated immediately before the final classifier. When metadata were excluded, the classifier received only the visual representation. The resulting vector $z_{i}^{\left(final\right)}$ was subsequently passed to the classifier in Equation (10), and the posterior class probabilities were obtained using Equation (11).

The compact projection network in Equation (8) was selected because the available metadata comprised four heterogeneous structured variables rather than a temporal sequence or free-text record. An Recurrent Neural Network (RNN) was not used because no sequential dependency existed among age, sex, anatomical site, and skin tone. A transformer-based metadata encoder could model interactions among these variables, but it would introduce additional parameterization for a four-variable input without a clear inductive advantage in the present setting. Since no controlled RNN- or transformer-based metadata experiment was conducted, the selected pathway is not claimed to be universally superior to these alternatives.

The final representation was forwarded to a fully connected classifier to produce logits for the lesion classes:

(10) $$o_{i}=f_{cls}\left(z_{i}^{\left(final\right)}\right),$$

In Equation (10), $f_{cls}\left(\cdot \right)$ denotes the final fully connected classifier, and $o_{i}\in R^{K}$ is the vector of class logits for lesion i, where K=11. The posterior class probabilities were obtained by softmax:

(11) $${\hat{p}}_{i}=softmax(o_{i}),{\hat{p}}_{i,k}=\frac{exp(o_{i,k})}{\sum_{j=1}^{K}exp(o_{i,j})}.$$

In Equation (11), ${\hat{p}}_{i,k}$ denotes the posterior probability assigned to class k for lesion i. The predicted class was determined as the class with the highest posterior probability. Unless otherwise stated, model training was performed using the standard multi-class cross-entropy loss

(12) $$L_{CE}=-\frac{1}{N}\sum_{i=1}^{N}log{\hat{p}}_{i,y_{i}},$$

which served as the primary optimization objective throughout the core experiments. This formulation was intentionally retained as the default learning setup to provide a stable and interpretable basis for the subsequent analyses of calibration, robustness, and explainability. Overall, the proposed framework combines modality-specific visual learning, sample-adaptive fusion, and metadata-aware contextual modeling within a single end-to-end architecture for clinically realistic 11-class skin lesion classification. The resulting logits and posterior probabilities formed the basis for all downstream analyses described in Section 3.3, including discrimination performance, calibration, robustness, and explainability assessment.

### 3.3. Experimental Design and Evaluation Strategy

The proposed framework was evaluated through a multi-stage experimental design intended to assess not only multi-class discrimination performance, but also confidence reliability, robustness under realistic image perturbations, and the clinical plausibility of model explanations. All experiments followed the lesion-level 3-fold group-wise cross-validation protocol described above. In each run, one fold was used for training, one for validation, and one for testing, ensuring that lesion identities never overlapped across subsets. Across the full protocol, each lesion appeared exactly once in the test set and once in the validation set. Classification, calibration, metadata-ablation, and paired statistical analyses were calculated from pooled out-of-fold predictions. Fold-level robustness results were summarized using the mean and standard deviation across the three outer folds. This evaluation design was intentionally constructed to assess not only predictive discrimination, but also the reliability and interpretability of the proposed framework under clinically relevant conditions.

Model training was performed end-to-end in PyTorch 2.5.1 on NVIDIA GeForce RTX 4070 GPUs. Optimization was carried out using the AdamW optimizer [42] with an initial learning rate of $1\times 10^{-4}$ and a weight decay of $1\times 10^{-4}$. A cosine annealing learning-rate schedule [43] was employed throughout training to improve convergence stability. The mini-batch size was set to 16, and all images were resized to 224×224 pixels. During training, data augmentation was restricted to the training split and consisted of random resized cropping and random horizontal flipping, whereas validation and test images were processed without stochastic augmentation. Early stopping was applied based on validation performance, and the checkpoint yielding the best validation result within each fold was retained for final evaluation. This strategy was adopted to balance optimization stability, computational efficiency, and protection against overfitting in the presence of class imbalance and limited minority-class samples.

Primary predictive performance was assessed on the clean test subsets using Accuracy, Macro F1-score, and Macro-Averaged AUC. Macro-averaged metrics were emphasized because they provide a more balanced view of performance under severe class imbalance and make degradation in underrepresented categories more visible than overall accuracy alone. To assess the contribution of individual metadata variables, a controlled ablation analysis was conducted using the gate-free dual-view configuration. The gate-free setting was selected to isolate the effect of metadata inclusion from variation introduced by adaptive fusion. In this configuration, the concatenated 3072-dimensional dual-view feature vector was passed through a learnable 3072-to-3072 linear projection, while the sample-adaptive gate was omitted. The visual encoders, lesion-level three-fold outer cross-validation partitions, training procedure, and evaluation protocol were kept unchanged. Six metadata settings were evaluated: no metadata, age only, sex only, anatomical site only, skin tone only, and all four variables. All metrics were calculated from pooled out-of-fold predictions. These comparisons were interpreted descriptively because paired significance tests were not performed for every ablation configuration.

In addition to discrimination performance, probability calibration was analyzed to determine whether model confidence was aligned with empirical correctness. Let ${\hat{p}}_{i,k}$ denote the predicted probability of class k for sample i, and let ${\hat{c}}_{i}={max}_{k}{\hat{p}}_{i,k}$ be the corresponding confidence. Expected Calibration Error (ECE) was computed over B confidence bins as

(13) $$ECE=\sum_{b=1}^{B}\frac{|I_{b}|}{N}|acc(I_{b})-conf(I_{b})|,$$

where $acc\left(I_{b}\right)$ and $conf\left(I_{b}\right)$ represent empirical accuracy and average confidence within bin $I_{b}$, respectively. Calibration quality was further quantified using Negative Log-Likelihood (NLL) and Brier Score, allowing confidence behavior to be examined from complementary perspectives.

(14) $$NLL=-\frac{1}{N}\sum_{i=1}^{N}log{\hat{p}}_{i,y_{i}}$$

It should be noted that the NLL in Equation (14) is mathematically equivalent to the cross-entropy loss defined in Equation (12). However, while Equation (12) serves as the training objective, NLL is retained here as a calibration diagnostic to quantify the average log-loss of the predicted probability distribution on held-out data.

(15) $$Brier=\frac{1}{N}\sum_{i=1}^{N}{∥{\hat{p}}_{i}-y_{i}∥}_{2}^{2}$$

where $y_{i}\in \{0,1{\}}^{K}$ denotes the one-hot encoding of the ground-truth label for sample i. To investigate whether post-hoc recalibration could improve probability estimates without altering decision boundaries, scalar temperature scaling [44] was applied on the validation fold according to

(16) $${\hat{p}}_{i}^{\left(T\right)}=softmax\left(\frac{o_{i}}{T}\right),$$

In Equation (16), T>0 denotes the scalar temperature parameter, and $o_{i}$ denotes the unscaled logit vector of sample i. For each outer fold, T was estimated exclusively from the corresponding validation logits by minimizing NLL and was subsequently applied without further optimization to the held-out test logits from that fold. After completion of the three outer folds, the raw and temperature-scaled test predictions were pooled separately to form complete out-of-fold prediction sets.

Calibration metrics and reliability diagrams were calculated from these pooled predictions. ECE was computed using 15 equal-width confidence bins. This procedure ensured that temperature estimation remained independent of the test observations while allowing calibration to be assessed over one held-out prediction for every lesion. The calibrated temperature obtained on the validation fold was subsequently used to recalibrate test-set logits, and reliability diagrams were generated for both validation and test predictions before and after temperature scaling. The multiclass Brier score was calculated as the mean sample-wise sum of squared differences between the predicted probability vector and the one-hot encoded class label across all 11 diagnostic categories.

Statistical comparisons among the principal model configurations were conducted using the pooled raw out-of-fold predictions. Since each lesion received one test prediction from each model, all comparisons were performed at the lesion level using paired procedures. Differences in Accuracy, Macro F1-score, and Macro-AUC were assessed using 2000 paired bootstrap resamples, from which 95% percentile confidence intervals and two-sided p-values were obtained. Paired permutation tests with 1000 permutations were additionally performed for the same metrics. For Accuracy, exact two-sided McNemar tests were applied to the discordant correct–incorrect prediction pairs. The analyses included comparisons between gated DVM-SLC and the gate-free metadata model, gated DVM-SLC and the dermoscopic-only model, and the gate-free metadata model and the dermoscopic-only model. Statistical significance was evaluated at p<0.05.

To characterize performance under class imbalance, diagnostic categories were divided into head and tail groups according to their mean test support across the three outer folds. Classes with a mean fold-specific test support of at least 30 were assigned to the head group, whereas classes below this threshold were assigned to the tail group. Class-specific F1-scores and one-versus-rest AUC values were calculated from the pooled out-of-fold predictions and were subsequently averaged with equal class weighting within each group. The head group comprised AKIEC, BCC, BKL, MEL, NV, and SCCKA, whereas the tail group comprised BEN_OTH, DF, INF, MAL_OTH, and VASC.

Robustness analysis was performed to assess the stability of the proposed framework under realistic acquisition-related perturbations. For this purpose, common image corruptions were applied simultaneously to both clinical and dermoscopic views at three increasing severity levels. The corruption set included Gaussian noise, blur, JPEG compression, brightness variation, contrast variation, and color shift. For a corruption type t and severity level s, the corrupted input pair was evaluated using the same trained model, and performance was recomputed independently for each setting. This procedure yielded a corruption-specific performance matrix for every fold. The same discrimination metrics used for clean testing, namely Accuracy, Macro F1-score, and Macro-Averaged AUC, were retained during robustness analysis to ensure comparability between clean and corrupted conditions. Let $m_{t,s}^{\left(f\right)}$ denote the value of a selected performance metric obtained in outer fold f under corruption type t and severity level s. For each corruption–severity combination, the fold-level mean and standard deviation were calculated as

(17) $$\mu_{t,s}=\frac{1}{F}\sum_{f=1}^{F}m_{t,s}^{\left(f\right)},\sigma_{t,s}=\sqrt{\frac{1}{F}\sum_{f=1}^{F}{\left(m_{t,s}^{\left(f\right)}\mu_{t,s}\right)}^{2}},$$

In Equation (17), F=3 denotes the number of outer folds, t identifies the corruption type, s identifies the severity level, and f denotes the outer-fold index. The term $m_{t,s}^{\left(f\right)}$ represents the fold-specific Accuracy, Macro F1-score, or Macro-AUC value, whereas $\mu_{t,s}$ and $\sigma_{t,s}$ denote the corresponding mean and standard deviation across the three folds.

Explainability analysis was conducted at both image and metadata levels. For image-based interpretation, Grad-CAM [3] was applied independently to the clinical and dermoscopic branches to identify the image regions contributing most strongly to the predicted class. Let A denote the activation tensor of a selected convolutional layer and $o_{\hat{y}}$ the logit corresponding to the predicted class $\hat{y}$. The Grad-CAM weights were obtained by spatially averaging the gradients of $o_{\hat{y}}$ with respect to the feature channels, and the resulting class activation map was computed as a weighted linear combination of feature maps followed by a ReLU operation.

(18) $$\alpha_{k}=\frac{1}{H^{\prime }W^{\prime }}\sum_{u=1}^{H^{\prime }}\sum_{v=1}^{W^{\prime }}\frac{\partial o_{\hat{y}}}{\partial A_{u,v,k}}$$

(19) $$CAM(u,v)=ReLU\left(\sum_{k=1}^{D}\alpha_{k}A_{u,v,k}\right).$$

In Equations (18) and (19), $A_{u,v,k}$ denotes the activation at spatial location (u, v) in feature channel k, and $o_{\hat{y}}$ denotes the logit associated with the predicted class $\hat{y}$. The coefficient $\alpha_{k}$ represents the spatially averaged gradient for channel k. The resulting class activation map was obtained as the rectified weighted sum of the feature channels and was subsequently resized to the input-image resolution.

To complement image-level interpretation, metadata contributions were analyzed using SHAP (version 0.49.1) [4]. In this setting, the metadata vector was treated as an auxiliary explanatory input, and feature attributions were estimated relative to a background distribution sampled from the training fold. This analysis enabled the contribution of variables such as age, sex, anatomical site, and skin tone to be examined at the feature level.

To evaluate the clinical relevance of the model’s attention, the Grad-CAM heatmaps generated for paired clinical and dermoscopic images were independently reviewed by one of the authors (R.Ö.K.), a dermatologist with 15 years of clinical experience in dermatology and dermoscopy. One representative case from each of the 11 diagnostic categories was examined using the original image, the corresponding Grad-CAM heatmap, and the available patient metadata. The evaluation considered whether the model correctly localized the lesion, including coverage of the lesion boundaries, attention to central and peripheral regions, and activation outside the lesion. In addition, the highlighted regions were assessed to determine whether they corresponded to clinically meaningful morphological and dermoscopic features, including vascular patterns, scales, erythema, alterations in the pigment network, blue-white structures, and other lesion-specific findings. The purpose of this evaluation was to determine whether the model’s attention was clinically meaningful rather than to measure segmentation performance. Therefore, quantitative localization metrics and interobserver agreement were not assessed.

Computational efficiency was evaluated under a common hardware and software environment using an NVIDIA GeForce RTX 4070 GPU and an input resolution of 224 × 224 pixels. For the dual-view configurations, one model input consisted of a paired clinical and dermoscopic image from the same lesion. Trainable parameters were counted for each configuration, and the number of floating-point operations was calculated as GFLOPs per lesion pair using fvcore. Checkpoint size was measured from the stored model file. Mean batch-1 inference latency and peak GPU memory usage were recorded during CUDA inference. These measurements were used for relative comparison among the evaluated configurations under the same computational conditions.

## 4. Results

In this study, the proposed model was evaluated not only in terms of overall classification performance, but also with respect to class-wise behavior, confidence reliability, robustness to image corruption, and expert-supported explainability. This broader evaluation was necessary because MILK10k represents a highly imbalanced 11-class problem, and a single summary metric may easily obscure clinically relevant weaknesses. For this reason, the findings are presented in four parts. First, comparative classification performance is examined. Then, reliability and robustness are assessed. Finally, the explainability results are discussed together with dermatologist-guided interpretation.

### 4.1. Comparative Classification Performance

Before examining class-wise behavior in more detail, a direct comparison among the evaluated model configurations was performed on the clean MILK10k test set. Such a comparison was particularly important because the proposed model departs from the baseline configurations through the combined use of dual-view learning, adaptive fusion, and contextual modeling rather than through a single design modification. Accordingly, an overall comparison was first used to show how these design choices were reflected in the main performance metrics, and the corresponding results are summarized in Table 1.

Except for clinical-only reference, all results were calculated from pooled raw out-of-fold predictions across the same three lesion-level cross-validation folds. The clinical-only values are those reported in the original submission and were retained as a descriptive reference. They were not included in the revised paired statistical analyses. For each delta column, the reference was the strongest non-proposed configuration evaluated under the revised out-of-fold protocol for the corresponding metric. Delta values were calculated from the unrounded results.

Among the configurations evaluated using pooled out-of-fold predictions, gated DVM-SLC achieved the highest Accuracy of 0.7002 and the highest observed Macro-AUC of 0.8774, whereas the gate-free dual-view model with metadata achieved the highest Macro F1-score of 0.4174. The Accuracy difference between gated DVM-SLC and the dermoscopic-only baseline, which achieved an Accuracy of 0.6874, was statistically significant. However, the observed Macro-AUC difference was not statistically significant. These findings indicated a metric-dependent trade-off rather than uniform superiority of a single model configuration. The corresponding paired statistical comparisons are presented in Table 2.

**Table 2:** Paired statistical comparisons among the principal model configurations based on pooled out-of-fold predictions.

Comparison	Metric	Difference ^a^	95% Bootstrap CI	Bootstrap (*p*)	Permutation (*p*)	McNemar (*p*)
Gated DVM-SLC − Gate-free dual-view + metadata	Accuracy	+0.0134	0.0019 to 0.0246	0.017	0.036	0.032
Gated DVM-SLC − Gate-free dual-view + metadata	Macro F1-score	−0.0415	−0.0664 to −0.0177	<0.001	0.004	—
Gated DVM-SLC − Gate-free dual-view + metadata	Macro-AUC	+0.0089	−0.0083 to 0.0260	0.284	0.376	—
Gated DVM-SLC − Dermoscopic-only	Accuracy	+0.0128	0.0013 to 0.0240	0.028	0.020	0.030
Gated DVM-SLC − Dermoscopic-only	Macro F1-score	−0.0013	−0.0247 to 0.0215	0.897	0.919	—
Gated DVM-SLC − Dermoscopic-only	Macro-AUC	+0.0087	−0.0083 to 0.0277	0.356	0.337	—
Gate-free dual-view + metadata − Dermoscopic-only	Accuracy	−0.0006	−0.0130 to 0.0116	0.972	0.963	0.952
Gate-free dual-view + metadata − Dermoscopic-only	Macro F1-score	+0.0401	0.0149 to 0.0644	0.001	0.003	—
Gate-free dual-view + metadata − Dermoscopic-only	Macro-AUC	−0.0002	−0.0191 to 0.0181	0.967	0.979	—

^a^ Differences were calculated as the first model minus the second model in each comparison.

Differences were calculated as the first model minus the second model in each comparison. Confidence intervals and p-values were calculated from pooled raw out-of-fold predictions. McNemar tests were applicable only to paired correctness outcomes and are therefore reported for Accuracy.

The paired analyses showed that the relative performance of the evaluated configurations depended on the selected metric. Compared with the dermoscopic-only baseline, gated DVM-SLC increased Accuracy by 0.0128, with a 95% bootstrap confidence interval of 0.0013–0.0240. This difference was supported by the paired bootstrap test (p=0.028), paired permutation test (p=0.020), and McNemar test (p=0.030). The corresponding differences in Macro F1-score and Macro-AUC were not statistically significant.

Compared with the gate-free dual-view model with metadata, gated DVM-SLC increased Accuracy by 0.0134 but reduced the Macro F1-score by 0.0415; both differences were statistically significant. The Macro-AUC difference between these configurations was not statistically significant. These findings show that gated fusion provided a modest advantage in overall classification accuracy, whereas the gate-free metadata model provided stronger class-balanced performance. No single configuration demonstrated statistically supported superiority across all evaluation metrics.

In Table 3, head classes were defined as categories with a mean test support of at least 30 across the three outer folds and included AKIEC, BCC, BKL, MEL, NV, and SCCKA. Tail classes had a mean test support below 30 and included BEN_OTH, DF, INF, MAL_OTH, and VASC. Group means were calculated by assigning equal weight to each class.

The limitation was particularly evident at the individual-class level. Within the tail group, the F1-scores were 0.0000 for BEN_OTH, 0.1563 for DF, 0.0755 for INF, 0.0000 for MAL_OTH, and 0.4578 for VASC. The complete failure to obtain a non-zero F1-score for BEN_OTH and MAL_OTH demonstrates that the aggregate performance of the model should not be generalized to all diagnostic categories.

The class-distribution analysis revealed a marked difference between the dominant and underrepresented categories. As shown in Table 3, the six head classes achieved a mean F1-score of 0.5743, whereas the mean F1-score of the five tail classes was 0.1379. The corresponding mean AUC values were 0.8968 and 0.8542. The smaller difference in AUC than in F1-score indicates that the model retained some one-versus-rest ranking information for the rare categories, but this information was not translated into reliable top-1 predictions. The higher tail AUC should therefore not be interpreted as evidence of satisfactory minority-class recognition. To examine the contribution of the individual metadata variables, the controlled ablation results are presented in Table 4.

Compared with the no-metadata configuration, using all four metadata variables left Accuracy almost unchanged, from 0.6872 to 0.6868, while increasing the Macro F1-score from 0.4079 to 0.4174, the Weighted F1-score from 0.6796 to 0.6847, and the Macro-AUC from 0.8370 to 0.8685. Among the single-variable configurations, age achieved the highest Macro-AUC of 0.8617, whereas anatomical site produced the highest Macro F1-score of 0.4062. Sex and skin tone alone yielded lower Macro F1-scores of 0.3334 and 0.3463, respectively. No individual variable reproduced the overall performance profile obtained when all four variables were combined. The contribution of metadata was therefore mainly reflected in class-balanced performance and discrimination rather than in overall Accuracy.

### 4.2. Reliability and Robustness Under Realistic Perturbations

**Figure 3:**
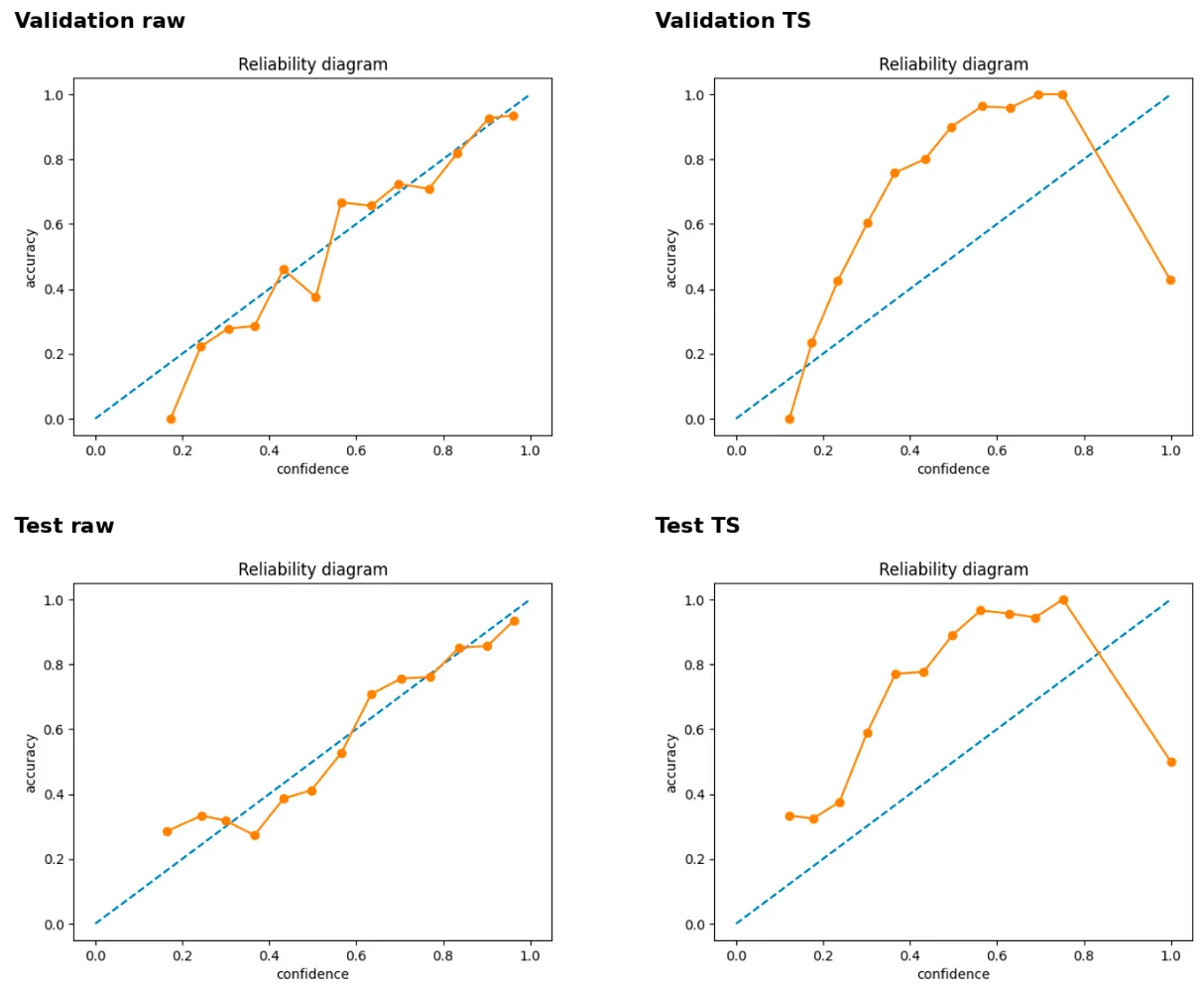
Reliability diagrams of DVM-SLC before and after temperature scaling on the validation and test sets. The blue dashed diagonal line represents perfect calibration, whereas the orange line with markers shows the empirical accuracy across confidence bins. TS denotes temperature scaling.

**Figure 4:**
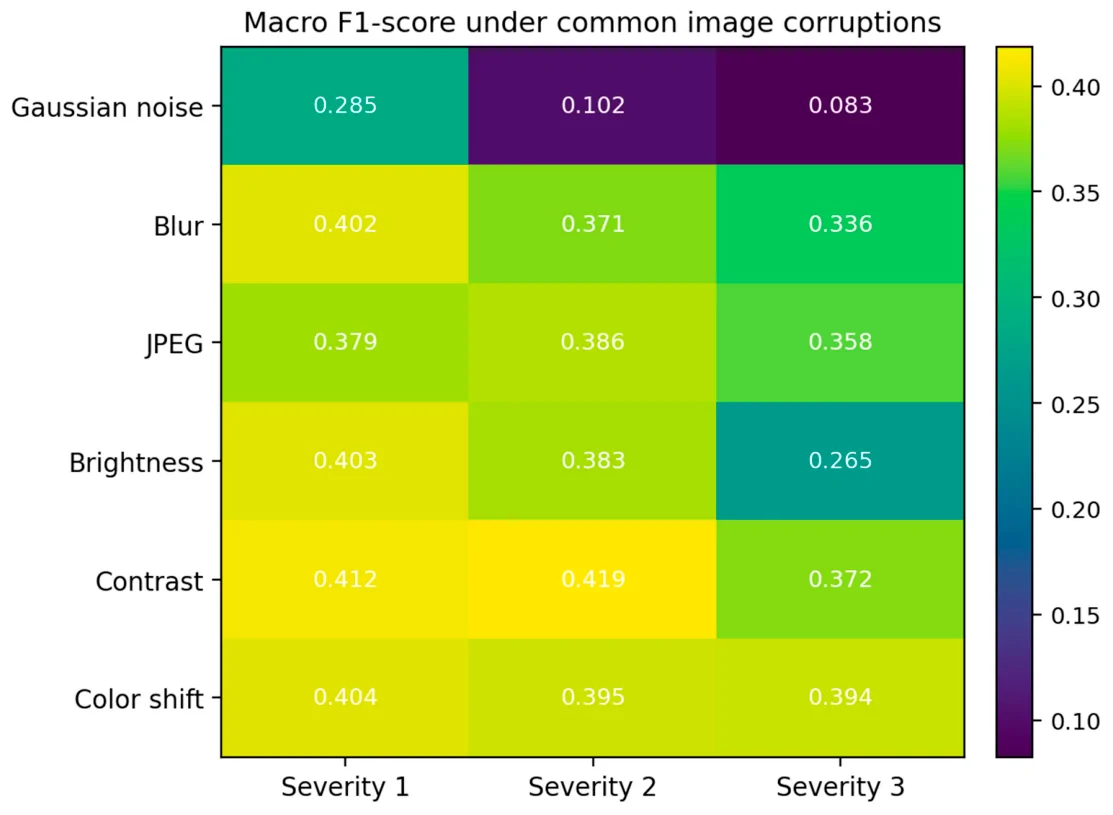
Severity-wise robustness of DVM-SLC under common image corruptions, reported in terms of Macro F1-score.

After the comparative classification results, the confidence behavior and robustness profile of DVM-SLC were examined to assess whether the model remained reliable beyond clean-set performance. This step was particularly important because, in medical image analysis, competitive classification scores alone do not necessarily indicate that model confidence is well behaved or that performance will remain stable under degraded image conditions. For this reason, calibration and corruption-based robustness were evaluated together, and the corresponding findings are given in Figure 3 and Figure 4 and Table 5 and Table 6. 

A separate scalar temperature was fitted on the validation logits of each outer fold and applied only to the corresponding held-out test logits. Calibration metrics were calculated after pooling the three sets of test predictions. ECE was calculated using 15 equal-width confidence bins. The raw out-of-fold predictions were already reasonably calibrated, with an ECE of 0.0365, an NLL of 0.9653, and a Brier score of 0.4344. Fold-specific temperature scaling produced small reductions in all three pooled measures, yielding an ECE of 0.0349, an NLL of 0.9504, and a Brier score of 0.4297. Since scalar temperature scaling does not alter the within-sample argmax, the predicted class labels and hard-decision metrics remained unchanged. At the fold level, calibration improved in folds 0 and 2 but deteriorated slightly in fold 1. The reliability diagrams in Figure 3 also showed greater under-confidence in parts of the intermediate confidence range after scaling. Temperature scaling was therefore interpreted as a modest, fold-dependent refinement rather than a uniform correction.

Corresponding Accuracy and Macro-AUC results across all severity levels are provided in Table 6.

The robustness analysis provides a similarly balanced view of model behavior. As shown in Figure 4 and Table 6, Gaussian noise produced the strongest and most consistent degradation across all reported metrics. This result was expected to some extent, since strong noise directly disrupts the fine structural and textural cues on which both clinical and dermoscopic analysis depend.

In contrast, the model remained considerably more stable under blur, JPEG compression, brightness variation, contrast variation, and color shift. Although these perturbations still caused a measurable decline, the reduction was clearly less severe than that observed under heavy noise contamination. This indicates that DVM-SLC is not uniformly fragile. Its main weakness is concentrated in severe stochastic degradation, whereas its behavior under more common acquisition-related variations remains comparatively stable.

This distinction is important for the interpretation of the model as a clinically oriented system. In many studies, performance is reported only under ideal test conditions, and the resulting numbers may therefore overestimate real-world readiness. In the present study, the model was examined under both clean and corrupted test conditions, making its strengths and limitations more visible. From this perspective, DVM-SLC can be described as a model with competitive clean-set performance, reasonably well-behaved raw confidence, and selective robustness rather than universal stability. This more explicit characterization also provides a stronger basis for the explainability analysis that follows.

### 4.3. Expert-Supported Explainability Analysis

**Figure 5:**
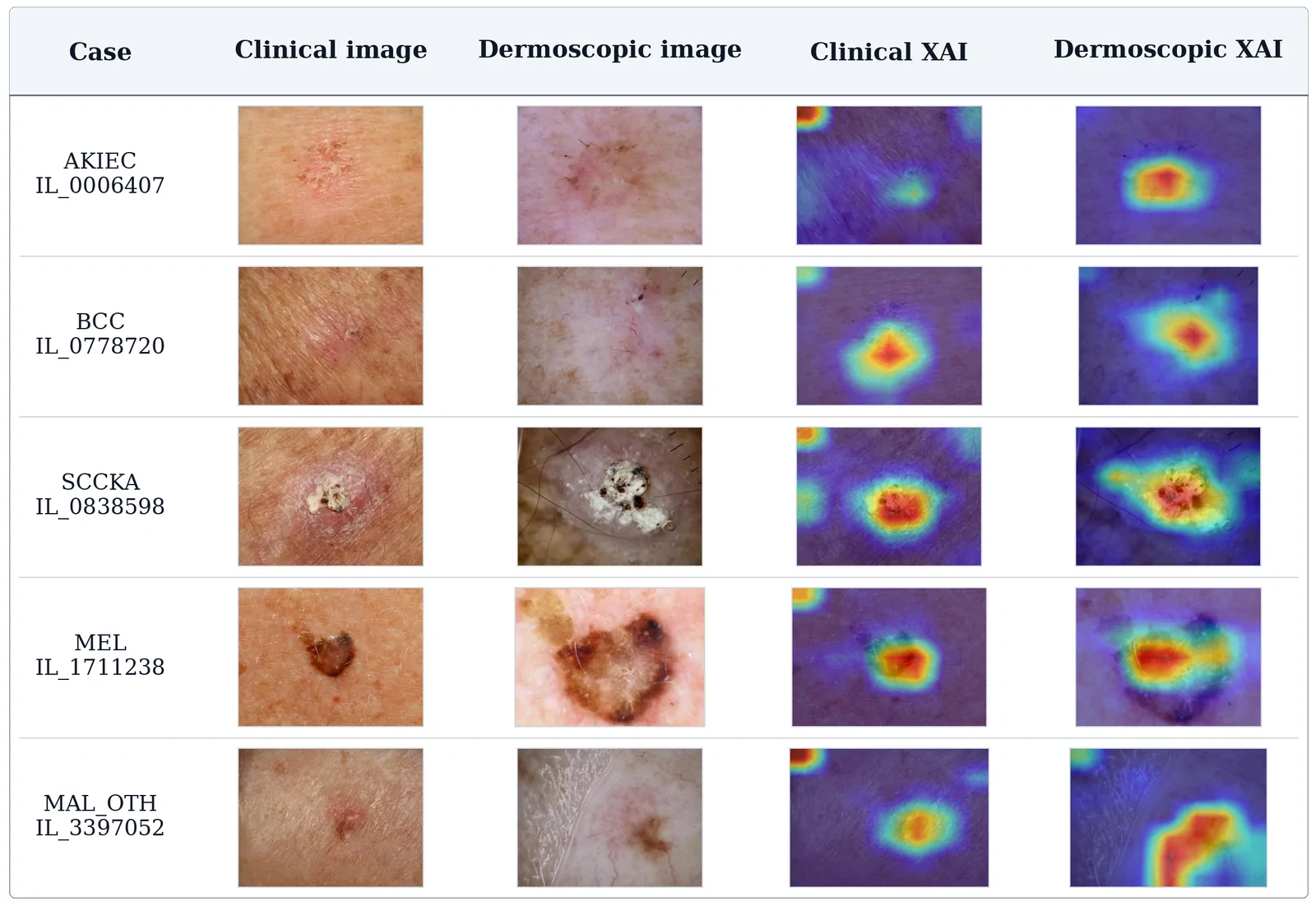
Expert-supported XAI examples from malignant and high-risk lesions. Each row presents the lesion type and identifier, followed by the clinical image, dermoscopic image, clinical XAI map (Grad-CAM), and dermoscopic XAI map (Grad-CAM). Lesion type abbreviations are defined in Section 3.1.

**Figure 6:**
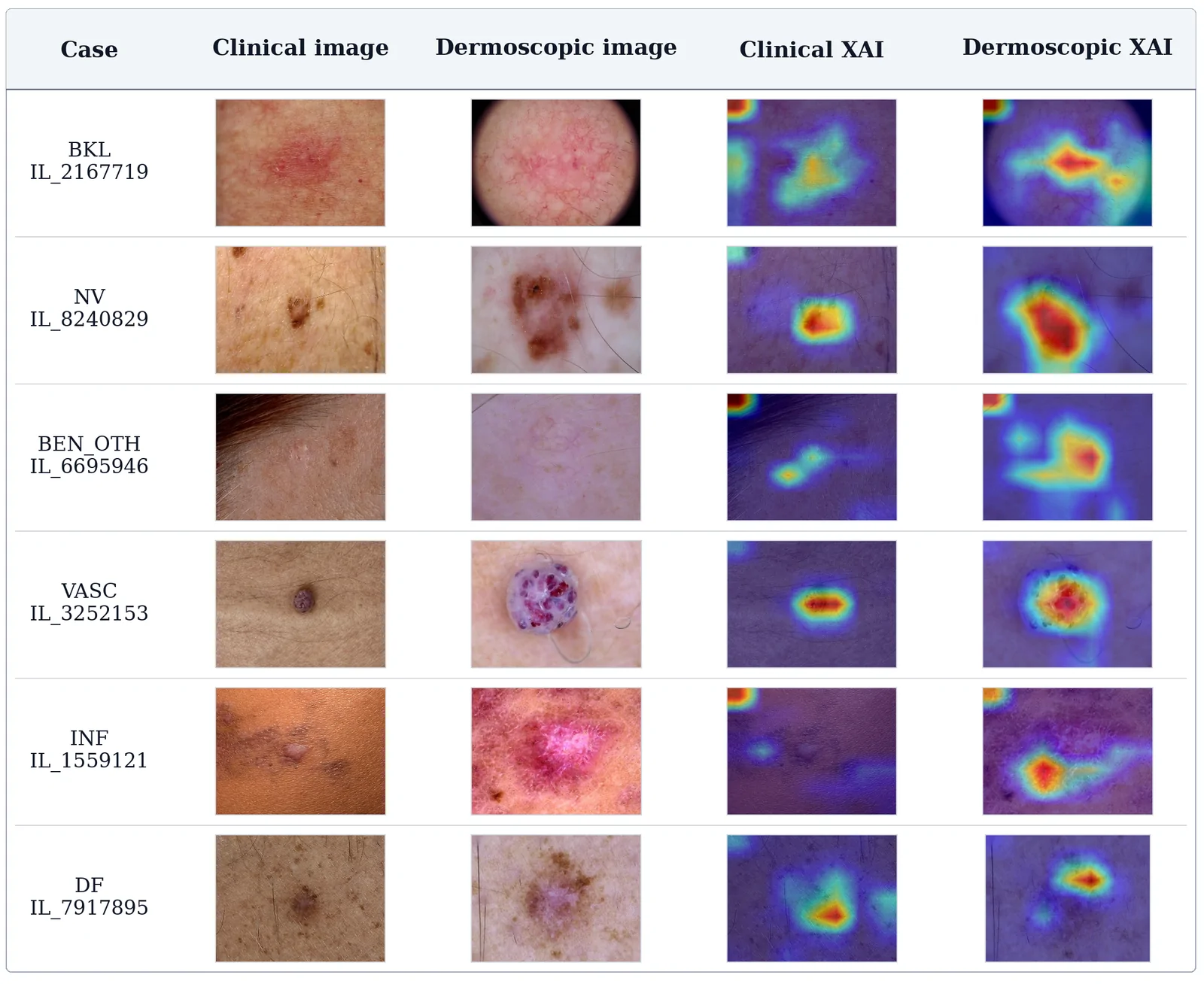
Expert-supported XAI examples from benign and non-malignant lesions. Each row presents the lesion type and identifier, followed by the clinical image, dermoscopic image, clinical XAI map (Grad-CAM), and dermoscopic XAI map (Grad-CAM). Lesion type abbreviations are defined in Section 3.1.

The clinical relevance of the Grad-CAM outputs was examined using the structured dermatologist review protocol described in Section 3.3. One representative case from each of the 11 diagnostic categories was evaluated using the paired clinical and dermoscopic images, the corresponding heatmaps, and the available patient metadata. The examples are presented in Figure 5 and Figure 6, and the case-specific interpretations are summarized in Table 7. These findings provide qualitative clinical context for the model attention but should not be interpreted as quantitative evidence of localization accuracy. In the Grad-CAM maps, warmer colors (yellow–red) indicate stronger relative class-discriminative activation, whereas cooler colors (blue–green) indicate weaker relative activation.

A consistent pattern emerged across the reviewed examples. In general, the dermoscopic branch produced more compact and lesion-centered attention, whereas the clinical branch tended to generate broader and less sharply bounded saliency regions. This difference was not uniform across all examples, but it was sufficiently recurrent to suggest that the two branches contributed different forms of information. The dermoscopic view was often more closely aligned with local morphological structures, while the clinical view appeared to capture a wider but sometimes less precise lesion context. This branch-specific behavior supports the rationale for dual-view modeling, since the two modalities appeared to provide complementary rather than redundant information.

The malignant and high-risk examples in Figure 5 illustrate this distinction clearly. In the AKIEC example, the dermatologist noted that the clinical XAI map did not adequately cover the superior pole of the lesion and failed to delineate the lesion as a whole, whereas the dermoscopic map captured only the central part. In the BCC example, the clinical branch was judged to have missed the lesion almost entirely, while the dermoscopic attention was considered directionally closer but still not ideally localized.

In the SCCKA example, the clinical map focused mainly on the lesion center and did not extend sufficiently toward the scaly peripheral areas, whereas the dermoscopic branch highlighted only part of the diagnostically relevant field. The MEL example provided an even stronger illustration of the value of expert review, since the clinical XAI failed to capture the protruding parts of the lesion and the dermoscopic map covered only a limited portion of the lesion. These examples show that visually plausible heatmaps do not necessarily imply clinically sufficient localization, and that expert review can reveal meaningful deficiencies that would otherwise remain hidden.

The benign and non-malignant examples in Figure 6 further strengthen this interpretation. In the BKL example, the clinical branch missed part of the lesion margin, whereas the dermoscopic branch was judged to be closer to an acceptable localization. In the NV example, the clinical map showed uneven lesion coverage, missing several sectors while overemphasizing others. In BEN_OTH and VASC, the clinical maps were considered weak or incorrect, while the dermoscopic branch provided a more meaningful focus, particularly in relation to crown vessels, clods, and vascular structures.

The INF and DF examples highlight another important point. In both, the dermatologist noted that the model failed to localize large portions of the lesion, especially in the clinical branch. Nevertheless, parts of the dermoscopic attention could still be related to meaningful clues such as scar-like depigmentation, chrysalis-like structures, asymmetric pigmented follicles, U-shaped vessels, and ring-like arrangements. These examples are scientifically valuable because they show that explainability should not be discussed only in successful predictions. In this study, partially correct or clinically insufficient attention patterns were also retained and interpreted. This makes the XAI analysis more honest and more informative.

The explainability results support three main conclusions. First, the two branches behaved differently in a clinically meaningful way, with the dermoscopic branch showing a more focused relation to lesion-specific structures in many examples. Second, expert review showed that visual plausibility alone is not sufficient for interpreting model attention, since apparently reasonable heatmaps may still miss diagnostically important parts of the lesion. Third, the analysis showed both clinically plausible attention patterns and cases in which attention remained incomplete or clinically insufficient. The findings therefore provide structured qualitative context for interpreting model attention, but their single-reviewer, case-based design limits the strength and generalizability of the conclusions.

### 4.4. Computational Efficiency

The computational characteristics of the principal model configurations are reported in Table 8.

As expected, the dermoscopic-only baseline was the most computationally efficient configuration because it used a single image encoder. Gated DVM-SLC contained 26.93 million trainable parameters and required 1.990 GFLOPs per lesion pair. Compared with the gate-free dual-view model with metadata, the gated configuration used approximately 15% fewer trainable parameters, had an approximately 15% smaller checkpoint, and required 4.6% less peak GPU memory. The nominal GFLOP counts of the two configurations were almost identical. The lower parameter count of Gated DVM-SLC reflects the implemented fusion layers. The gate-free configuration applies a full 3072-to-3072 linear projection to the concatenated dual-view vector, whereas the gated configuration uses a 3072-to-1536-to-1 gating network. However, the batch-1 inference latency of gated DVM-SLC was 29.98 ms, compared with 26.68 ms for the gate-free model. Gated fusion therefore reduced model size and memory usage without increasing the nominal operation count, although it introduced a modest latency increase.

## 5. Discussion

The present study examined multi-class skin lesion classification from a broader perspective than is commonly seen in the recent literature. Rather than limiting the analysis to aggregate classification performance, the proposed framework was evaluated with respect to class-wise behavior, calibration, robustness under image corruption, and expert-supported explainability. This evaluation design was particularly important because MILK10k represents a highly imbalanced 11-class problem in which a single summary metric can conceal important weaknesses.

The divergence between Accuracy and Macro F1-score is consistent with the severe class imbalance in MILK10k. Accuracy is influenced more strongly by the frequent categories, whereas Macro F1-score gives equal weight to every diagnostic class. The improved Accuracy of Gated DVM-SLC therefore indicates a benefit in overall correct classification, while the higher Macro F1-score of the gate-free metadata model indicates more balanced class-wise behavior. This pattern suggests that sample-adaptive fusion benefited prevalent diagnostic patterns more than the tail categories. Gated fusion should therefore be considered an operating-objective-dependent design choice rather than a uniformly superior mechanism. In a clinical decision-support setting, prioritizing overall correctness and prioritizing balanced performance across diagnostic categories may lead to different model choices.

Computational analysis adds a practical dimension to this comparison. The dual-view configurations required approximately twice the nominal operation count of the dermoscopic-only baseline because two image encoders were used. Within the dual-view setting, gated DVM-SLC contained fewer trainable parameters and required less model storage and peak GPU memory than the gate-free metadata model. This reduction was accompanied by a modest increase in batch-1 inference latency. The gated architecture should therefore be interpreted as a trade-off between adaptive fusion, model size, and latency rather than as a uniformly more efficient configuration.

The observed fusion behavior should also be interpreted in relation to recent state-of-the-art multimodal formulations. FusionM4Net combines feature- and decision-level fusion [33], SkinM2Former employs tri-modal cross-attention across clinical, dermoscopic, and metadata streams [36], and more recent studies have explored self-supervised paired-view learning [37], cross-attention with structured metadata [38], and uncertainty-guided adaptive fusion [39]. Vision–language encoders such as MedSigLIP represent an additional direction based on large-scale image–text pretraining [40]. In contrast, DVM-SLC adopts a more direct gated late-fusion design and preserves the original numerical and categorical representation of the structured metadata. Its contribution, therefore, lies not in claiming architectural superiority but in providing a leakage-safe, lesion-level evaluation of paired clinical and dermoscopic images, together with structured metadata, in the imbalanced 11-class MILK10k setting. This evaluation further incorporates calibration, robustness to corruption, metadata ablation, computational analysis, and dermatologist-supported explainability. Because the cited studies differ in their datasets, label spaces, available modalities, and evaluation protocols, their reported scores are not directly comparable. Matched benchmarking under a common experimental protocol remains necessary.

The head–tail analysis identifies minority-class recognition as a central limitation of the framework. Although the one-versus-rest AUC values suggest that some discriminatory ordering was retained for the rare categories, the low F1-scores show that this information was insufficient for dependable class assignment. This distinction is clinically important because ranking ability alone does not ensure that an underrepresented lesion will be assigned to the correct diagnostic category. The zero F1-scores observed for two tail classes further demonstrate that aggregate Accuracy and Macro-AUC cannot be used as evidence of uniform diagnostic reliability. The marked head–tail gap may reflect both limited training support and visual overlap between rare and more frequent categories. The present analysis cannot separate these effects, and larger rare-class cohorts would be required to determine their relative influence. More effective long-tail learning, larger rare-class cohorts, and class-sensitive decision strategies are therefore required before dependable recognition of the full diagnostic spectrum can be claimed.

The calibration analysis showed that the raw probability estimates were reasonably aligned with observed correctness at the aggregate level. Temperature scaling yielded small reductions in pooled ECE, NLL, and Brier score, but the effect was not consistent across folds, and greater under-confidence was observed in parts of the intermediate confidence range. It should therefore be regarded as modest, fold-dependent refinement rather than a uniformly beneficial correction. The low global ECE should also be interpreted cautiously because it is sensitive to binning and class prevalence and may conceal class-specific calibration errors in a severely imbalanced multi-class setting. Accordingly, the present results support only an aggregate calibration interpretation and do not establish uniformly reliable confidence estimates for every diagnostic category.

The robustness analysis also contributes to a more realistic reading of model behavior. As expected, Gaussian noise caused the strongest degradation across all reported metrics, showing that stochastic disruption of fine structural and textural information remains a major vulnerability for both clinical and dermoscopic analysis. In contrast, the model was more stable under blur, JPEG compression, brightness variation, contrast variation, and color shift. This distinction is meaningful because it suggests that the framework is not uniformly fragile. Its weaknesses are concentrated under severe noise contamination rather than spread evenly across all perturbation types. This selective robustness is more informative than a simple claim of stability, since it identifies the conditions under which performance is more likely to degrade and therefore provides a more realistic basis for future refinement.

The structured dermatologist review provided clinical context for interpreting the Grad-CAM outputs. Across the representative cases, the dermoscopic branch often produced more compact and lesion-centered attention, whereas the clinical branch more frequently captured a broader contextual field. These branch-specific patterns are consistent with the complementary roles of the two imaging modalities. Clinical images provide lesion-scale and surrounding context, whereas dermoscopic images reveal more localized morphological structures. However, the qualitative review does not establish how the model internally represents or uses these features. Several heatmaps highlighted clinically meaningful features but showed incomplete lesion coverage or missed diagnostically important structures. These observations illustrate both the potential and the limitations of model attention at the case level. However, the assessment was qualitative and was conducted by one dermatologist on one representative case per diagnostic category. The findings therefore should not be interpreted as quantitative localization validation or as evidence of general clinical reliability.

When the present findings are viewed in relation to literature, three broader implications emerge. First, strong aggregate metrics should be interpreted cautiously in imbalanced multi-class settings, since they may conceal poor rare-class commitment. Second, architectural sophistication alone does not guarantee better classification performance, as illustrated by the difference between the gated and no-gate dual-view settings. Third, explainability gains credibility only when the computational outputs are examined against domain expertise. These points position the present study less as a claim of absolute model superiority and more as a carefully structured analysis of how a dual-view skin lesion classifier behaves under realistic conditions. Such a contribution is methodologically valuable, particularly in a field where reliable deployment depends not only on classification accuracy, but also on confidence quality, perturbation sensitivity, and clinical plausibility.

Several limitations constrain the interpretation of the present findings. First, although MILK10k is a multi-center, clinically realistic dataset, all model development and internal validation were performed on a single dataset. Consequently, the reported findings on classification, calibration, robustness, and qualitative explainability should not be interpreted as evidence of external generalizability or clinical readiness. Validation on an independent cohort would be necessary to determine whether these findings remain stable across acquisition settings, institutions, devices, and patient populations. In addition, controlled experimental comparisons with more recent visual backbones, multimodal transformers, cross-attention models, and medical image–text encoders, such as MedSigLIP, were not conducted. Such comparisons would require identical lesion-level partitions, label definitions, input modalities, and downstream training conditions. Matched evaluation of these architectures under the same MILK10k protocol is therefore left for future investigation.

Second, the controlled metadata ablation showed that no individual variable reproduced the combined performance profile obtained when all four metadata variables were used. Age yielded the highest single-variable Macro-AUC, whereas anatomical site produced a Macro F1-score closest to that of the no-metadata configuration. The results indicate that the variables provided complementary information, mainly through improvements in class-balanced performance and discrimination rather than overall Accuracy. However, these comparisons remain descriptive because paired significance tests were not performed for every ablation configuration.

Third, the gated and gate-free metadata models exhibited a metric-dependent trade-off. The gated model achieved higher Accuracy and the highest observed Macro-AUC, whereas the gate-free model achieved higher Macro F1 and Weighted F1 scores. Future work should therefore investigate when adaptive fusion improves overall correct classification and when the gate-free configuration better supports class-balanced performance.

Finally, the XAI assessment was conducted by one dermatologist on one representative case per diagnostic category. Pixel-level expert segmentation masks were not available, and quantitative localization metrics could therefore not be calculated. Evaluation by multiple independent experts and interobserver agreement analysis were also beyond the scope of the present study. The XAI findings should consequently be interpreted as structured qualitative observations rather than quantitative validation of localization performance. Calibration was evaluated globally, and class-conditional calibration could not be estimated reliably for the rarest categories because of their limited sample sizes. Computational efficiency was measured on a single NVIDIA GeForce RTX 4070 GPU, and the reported latency and memory values may vary across hardware platforms, software environments, and deployment settings.

Future work can therefore proceed in several complementary directions. External validation should be prioritized to test whether the observed performance profile remains stable beyond MILK10k. In addition, imbalance-aware optimization and class-sensitive decision strategies may help improve tail-class commitment without sacrificing the ranking structure that appears to be partially preserved even in difficult minority categories. On the architectural side, the present results suggest that future fusion designs should be compared not only in terms of flexibility, but also in terms of when and how that flexibility translates into measurable benefit. Future explainability studies should include pixel-level expert annotations, assessment by multiple independent dermatologists, and interobserver agreement analysis to enable quantitative evaluation of localization performance.

Overall, the findings do not support uniform dominance of a single fusion strategy. Gated DVM-SLC provided a statistically supported improvement in Accuracy and the highest observed Macro-AUC, whereas the gate-free metadata model retained the strongest Macro F1 and Weighted F1 performance. The contribution of the study is therefore framed as a transparent evaluation of metric-dependent fusion behavior together with reliability, robustness, and explainability, rather than as an assertion that the proposed architecture is superior under every evaluation criterion. The study therefore provides a structured basis for interpreting the classification performance, confidence behavior, perturbation sensitivity, and qualitative attention patterns of a dual-view skin lesion classifier.

## 6. Conclusions

This study examined multi-class skin lesion classification from paired clinical and dermoscopic images using a lesion-level evaluation protocol that included discrimination, calibration, robustness, and qualitative explainability assessment. A dual-view meta-aware framework, termed DVM-SLC, was developed to integrate modality-specific visual representations with patient metadata and to support not only discrimination analysis, but also calibration, robustness, and expert-supported explainability. The results showed that the effect of fusion strategy depended on the selected evaluation criterion. Gated DVM-SLC achieved the highest Accuracy and the highest observed Macro-AUC, whereas the gate-free metadata model achieved the highest Macro F1-score and Weighted F1-score. The Accuracy advantage of the gated model over the strongest matched comparator was statistically significant, while a significant Macro-AUC advantage was not established. These findings demonstrate a trade-off between overall correct classification and class-balanced performance rather than uniform superiority of either fusion strategy.

The results also showed that aggregate classification scores alone provide an incomplete account of model behavior. The head–tail analysis made the effect of class imbalance explicit, showing that minority classes remained substantially more difficult than dominant categories. The calibration results indicated that the raw confidence outputs were already reasonably aligned with empirical correctness, while temperature scaling provided only modest, fold-dependent improvements in aggregate calibration. Robustness analysis further showed that the framework was not uniformly fragile but was particularly vulnerable to severe Gaussian noise while remaining comparatively stable under several common acquisition-related perturbations. These findings make the reported performance profile more transparent and more clinically interpretable.

Minority-class performance constitutes an additional limitation. In particular, the absence of non-zero F1-scores for BEN_OTH and MAL_OTH limits the suitability of the current model for settings in which dependable recognition of rare diagnostic categories is required. The reported aggregate metrics should therefore be interpreted as an overall performance profile rather than as evidence of reliable performance for every class.

The Grad-CAM outputs of the clinical and dermoscopic branches were examined through a structured dermatologist review. The representative cases indicated that the dermoscopic branch often produced more lesion-centered attention, whereas the clinical branch more frequently captured broader contextual information. Several visually plausible heatmaps nevertheless showed incomplete lesion coverage or missed clinically relevant structures. Because the assessment was qualitative and was conducted by one dermatologist, these findings provide case-level clinical context but do not constitute quantitative localization validation.

Overall, the contribution of this work lies not only in proposing a dual-view skin lesion classification model, but also in presenting a more transparent evaluation framework for understanding how such models behave under realistic conditions. In this respect, the study offers a more complete basis for interpreting performance, confidence, robustness, and clinical plausibility in multi-class skin lesion analysis. Future work should focus on external validation, stronger strategies for tail-class improvement, and larger-scale expert-supported explainability protocols to further strengthen the clinical relevance of the proposed framework.

## Figures and Tables

**Table 1:** Overall classification performance of the evaluated model configurations on clean MILK10k data.

Model	Input Setting	Fusion Strategy	Metadata	Accuracy	Macro F1-Score	Weighted F1-Score	Macro-AUC	Δ Macro F1 vs. Best Baseline	Δ Macro-AUC vs. Best Baseline
Clinical-only baseline	Clinical image only	None	No	0.430	0.257	0.466	0.763	-	-
Dermoscopic-only baseline	Dermoscopic image only	None	No	0.6874	0.3773	0.6706	0.8687	-	-
Dual-view baseline	Clinical + dermoscopic	Concatenation + linear projection	No	0.6872	0.4079	0.6796	0.8370	−0.0095	−0.0317
Dual-view + metadata (no gate)	Clinical + dermoscopic + metadata	Concatenation + linear projection	Yes	0.6868	**0.4174**	**0.6847**	0.8685	0.000	−0.0002
Proposed DVM-SLC	Clinical + dermoscopic + metadata	Gated fusion	Yes	**0.7002**	0.3759	0.6779	0.8774	−0.0415	+0.0087

Bold values indicate the highest value obtained for each performance metric.

**Table 3:** Head–tail performance of gated DVM-SLC based on pooled out-of-fold predictions.

Group	No. Classes	Pooled OOF Support	Mean F1-Score	Mean AUC
Head classes	6	5038	0.5743	0.8968
Tail classes	5	202	0.1379	0.8542

**Table 4:** Controlled metadata ablation results for the gate-free dual-view model based on pooled out-of-fold predictions.

Metadata Setting	Accuracy	Macro F1-Score	Weighted F1-Score	Macro-AUC
No metadata	0.6872	0.4079	0.6796	0.8370
Age only	0.6840	0.3735	0.6716	0.8617
Sex only	0.6777	0.3334	0.6477	0.8504
Anatomical site only	0.6805	0.4062	0.6712	0.8487
Skin tone only	0.6756	0.3463	0.6512	0.8522
All metadata	0.6868	0.4174	0.6847	0.8685

**Table 5:** Calibration performance of gated DVM-SLC based on pooled out-of-fold predictions.

Setting	ECE	NLL	Brier Score
Raw predictions	0.0365	0.9653	0.4344
Temperature-scaled predictions	0.0349	0.9504	0.4297

**Table 6:** Severity-averaged robustness results under common image corruptions.

Condition	Accuracy	Macro F1-Score	Macro-AUC
Gaussian noise	0.326	0.156	0.671
Blur	0.698	0.370	0.860
JPEG compression	0.669	0.375	0.832
Brightness variation	0.663	0.350	0.837
Contrast variation	0.716	0.401	0.840
Color shift	0.715	0.398	0.868

**Table 7:** Dermatologist-guided interpretation of representative XAI cases.

Case/Lesion Type	Clinical Branch Assessment	Dermoscopic Branch Assessment	Key Clinical/Dermoscopic Cues Identified by the Dermatologist	Overall Interpretation
AKIEC (IL_0006407)	Incomplete lesion coverage; superior pole missed.	Central localization only; full lesion not captured.	Erythema, white-yellow scale, linear/dotted/corkscrew vessels, brown annular structure.	Clinically meaningful cues were present, but attention remained incomplete in both branches.
BCC (IL_0778720)	Lesion largely missed.	Directionally closer, yet still suboptimal.	Branching vessels, structureless area, brown annular pattern.	Dermoscopic attention was more informative, but localization was still clinically insufficient.
SCCKA (IL_0838598)	Mainly central focus; peripheral scaly area underrepresented.	Highlighted regions remained incomplete.	Dotted vessels, pseudofollicular openings, white scale, crust.	The lesion contained clear diagnostic clues, but branch attention did not fully cover them.
MEL (IL_1711238)	Protruding margins missed; boundaries poorly defined.	Only part of the lesion captured.	Blue-white structures, fissure-ridge pattern, fingerprint-like structures.	A visually plausible map did not translate into clinically sufficient lesion coverage.
MAL_OTH (IL_3397052)	Relatively close to appropriate localization.	Partial lesion coverage with some non-lesional focus.	Curved branching vessels, dotted vessels, ring-in-ring pattern.	One of the stronger examples overall, although the dermoscopic attention was still not fully confined to the lesion.
BKL (IL_2167719)	Part of the lesion margin missed.	Closer to acceptable localization.	Dotted, corkscrew, and branching vessels with scale.	Dermoscopic attention aligned better with lesion structure than the clinical branch.
NV (IL_8240829)	Uneven coverage with sector-wise omissions and overemphasis.	More lesion-centered than the clinical branch.	Forked and reticular vessels, focal hypopigmentation, atypical pigment network, concentric structures.	The example highlights the complementarity of the two branches rather than simple agreement.
BEN_OTH (IL_6695946)	Clinically weak and poorly localized.	Poor localization overall, although some focal saliency was present.	Coarse white-yellow clods, crown vessels.	A difficult example in which expert review was necessary to clarify which clinically relevant structures were not adequately captured by the model.
VASC (IL_3252153)	Spatial orientation inconsistent with the lesion.	More meaningful lesion focus than the clinical branch.	Clod-like vascular structures.	The dermoscopic branch provided more clinically relevant attention.
INF (IL_1559121)	Large parts of the lesion missed.	Only the lower half was captured.	Scar-like depigmentation, chrysalis-like structures, asymmetric pigmented follicles.	The attention pattern was partially correct and clinically interpretable yet still remained incomplete.
DF (IL_7917895)	Clinically uninformative focus.	Only the upper third was captured.	Scar-like depigmentation, asymmetric pigmented follicles, U-shaped vessels, white ring structures.	A representative example in which dermoscopic attention remained interpretable despite incomplete lesion coverage.

**Table 8:** Computational efficiency of the principal model configurations under the same GPU environment.

Model Configuration	Trainable Parameters (M)	GFLOPs per Lesion Pair	Checkpoint Size (MB)	Batch-1 Latency (ms)	Peak GPU Memory (MB)
Dermoscopic-only baseline	11.12	0.993	42.92	14.43	310.19
Dual-view baseline	31.65	1.994	121.74	27.06	389.11
Dual-view + metadata (no gate)	31.65	1.994	121.76	26.68	389.42
Gated DVM-SLC	26.93	1.990	103.76	29.98	371.42

## Data Availability

The data presented in this study are available in the ISIC Archive at https://doi.org/10.34970/648456, reference number 10.34970/648456. These data were derived from the following resource available in the public domain: the MILK10k dataset, https://api.isic-archive.com/doi/milk10k/ (accessed on 1 January 2026).

## Abbreviations

The following abbreviations are used in this manuscript:

AI	Artificial Intelligence
AKIEC	Actinic Keratosis/Intraepithelial Carcinoma
AUC	Area Under the Receiver Operating Characteristic Curve
BCC	Basal Cell Carcinoma
BEN_OTH	Other Benign Lesions
BKL	Benign Keratosis-Like Lesions
CNN	Convolutional Neural Network
DF	Dermatofibroma
DVM-SLC	Dual-View Meta-Aware Skin Lesion Classifier
ECE	Expected Calibration Error
GAP	Global Average Pooling
Grad-CAM	Gradient-weighted Class Activation Mapping
INF	Inflammatory Lesions
ISIC	International Skin Imaging Collaboration
MAL_OTH	Other Malignant Lesions
MEL	Melanoma
MILK10k	Multimodal and Multi-center Skin Lesion Dataset (MILK10k)
NLL	Negative Log-Likelihood
NV	Melanocytic Nevus
ReLU	Rectified Linear Unit
RNN	Recurrent Neural Network
SCCKA	Squamous Cell Carcinoma/Keratoacanthoma
SHAP	SHapley Additive exPlanations
VASC	Vascular Lesions
XAI	Explainable Artificial Intelligence
